# A study on the classification and prediction of firefighter’s operational fatigue level

**DOI:** 10.1371/journal.pone.0323911

**Published:** 2025-05-15

**Authors:** Mingwei Xu, Shangxue Yang, Ke Wang, Chengliu Yu, Guanlin Liu, Chao Dai, Ruiqi Wang

**Affiliations:** 1 School of Safety Engineering, Beijing Institute of Petrochemical Technology, Beijing, China; 2 National Center for Occupational Safety and Health, Beijing, China; 3 School of Engineering and Technology, China University of Geosciences (Beijing), Beijing, China; Louisiana State University Health Sciences Center Shreveport: LSU Health Shreveport, UNITED STATES OF AMERICA

## Abstract

Firefighting operations in high-rise building fires require firefighters to navigate complex environments while undertaking physically demanding, heavy-load tasks, which often lead to severe fatigue, impairing their operational efficiency and decision-making. This study aims to develop a robust fatigue classification and prediction model to assess and forecast firefighters’ fatigue levels. Key metrics, including electrocardiogram (ECG) signals, subjective fatigue ratings, and reaction time data, were utilized. Experiments involving six healthy adult male participants simulated firefighting scenarios, during which subjective fatigue levels (6–20 Borg’s RPE scale) and reaction times were recorded. A five-level fatigue classification was established using K-means clustering, and entropy weight analysis was applied to define a comprehensive fatigue index (F), enabling a three-tier fatigue classification: light, moderate, and severe fatigue. A BP neural network was employed for dynamic fatigue prediction, with 10 features derived from heart rate and heart rate variability (HRV) metrics serving as inputs and the comprehensive fatigue index (F) as the output. The BP neural network model achieved a high prediction accuracy with an R² value of 93.24%, demonstrating its capability to accurately predict firefighters’ fatigue states. This approach provides a scientific basis for optimizing firefighter training protocols and enhancing operational effectiveness during fire rescue missions. The findings highlight the significant potential of this method for advancing firefighter fatigue monitoring and management.

## 1. Introduction

According to statistics from the National Fire and Rescue Administration (January–October 2024), fire and rescue teams nationwide responded to 770,000 fire suppression incidents, resulting in 1,559 fatalities, a 12% increase compared to the previous year [1]. These figures underscore the intense demands and risks inherent in firefighting operations. During fire suppression, firefighters face complex environments and high-intensity tasks (e.g., climbing with heavy loads), which can rapidly induce fatigue. Studies demonstrate that fatigue significantly reduces operational efficiency and decision-making accuracy: Dennison et al. found that when firefighters’ heart rates exceeded 85% of their maximum, task completion time increased by 40%, and error rates rose to 28% [2]. Similarly, Yadav et al. reported a 35% increase in diagnostic errors and significant decision-making delays (p < 0.01) among medical staff working 12-hour shifts [3]. These findings highlight the universal impact of fatigue in dynamic operational contexts.

Existing research reveals fatigue’s broad effects on performance: Rangan et al. observed that fatigued pilots exhibited 2.3-fold higher risk assessment biases compared to their rested state [4]. Forte et al. demonstrated a strong correlation (r = 0.72, p < 0.001) between autonomic nervous system imbalance (via ECG parameters) and cognitive decline [5]. While fatigue detection models based on physiological signals (e.g., voice, EEG) achieve high accuracy in static environments [6–7], their applicability to dynamic firefighting scenarios remains unvalidated. Although machine learning models (e.g., BP neural networks) have shown promise in contexts like driving and healthcare [6–7], their reliance on single physiological indicators (e.g., heart rate variability) or subjective scales limits their suitability for the multidimensional demands of firefighting.

To address these gaps, this study proposes a dynamic fatigue classification and prediction framework that integrates firefighters’ electrocardiogram (ECG) signals, Borg RPE scores, and reaction time data. ECG provides objective insights into autonomic nervous system dynamics [5]; the Borg RPE scale, a globally validated tool, correlates strongly with physiological load [8]; and reaction time directly quantifies declines in attention and response speed [9]. Together, these metrics holistically capture fatigue across physiological, subjective, and cognitive dimensions, overcoming the limitations of single-data-source approaches. Through cluster analysis, fatigue levels are classified, a composite index is constructed to quantify fatigue severity, and a neural network model is developed for real-time prediction, aiming to enhance firefighter safety through precision monitoring.

## 2. Method

### 2.1. Participants

- 6 Six healthy adult males with a long-term commitment to physical fitness were selected for this study. The inclusion criteria for good physical fitness were as follows:
- Regular participation in aerobic or mixed training (≥4 sessions per week, each lasting ≥45 minutes) for at least two years.
- Meeting physical fitness standards: VO₂max ≥45 mL/kg/min, ≥40 sit-ups per minute, BMI 18.5–24.9 kg/m², no history of cardiovascular disease, resting heart rate 50–90 bpm, and blood pressure <140/90 mmHg.

Participants were restricted to males to control for potential confounding effects of gender-related physiological differences (e.g., menstrual cycle variations in fatigue perception and cardiovascular response) and to align with the demographics of frontline firefighters in the target population, where males constitute over 95% of personnel [National Fire Protection Association, 2023].Detailed participant information is provided in Table 1. The average age was 23.7 years, with each participant engaging in physical activity 3–4 days per week. All participants were mentally healthy, as confirmed by the PHQ-9 depression scale (score <5) and GAD-7 anxiety scale (score <5). They reported no issues with insomnia, late-night habits, or excessive alcohol consumption, and had no physical mobility impairments. To minimize the influence of external factors on experimental outcomes, participants were required to abstain from alcohol for 48 hours prior to the experiment (verified via breathalyzer) and maintain a sleep duration of ≥ 7 hours per night (monitored using wrist-worn sleep trackers). Before the experiment, all participants were thoroughly briefed on the procedures and overall process to ensure smooth execution.This study adhered to the principles of the Declaration of Helsinki and was approved by the Ethics Committee of Beijing Institute of Petrochemical Technology. All participants provided written informed consent, which included details of the experimental purpose, procedures, risk disclosure, and privacy protection clauses. Participants retained the right to withdraw unconditionally at any time, and their data were used solely for this research.A priori power analysis was conducted using G*Power 3.1 to determine the minimum sample size required for detecting a medium effect size (Cohen’s f² = 0.25) with 80% power at α = 0.05. The analysis indicated a required sample size of 6 participants, aligning with the inclusion of six participants in this study.

**Table 1:** Demographic information table of the experimental subject.

Number	Age	Height	Weight	Body Mass Index	Weight level	Exercise frequency
1	24	184	82	24.2	Overweight	5/7
2	25	173	53	17.7	Low weight	4/7
3	23	180	65	20.1	Normal	3/7
4	23	178	75	23.7	Normal	3/7
5	23	178	74	23.4	Normal	4/7
6	24	179	62	19.4	Normal	5/7
Mean value	23.67	178.67	68.5	21.42	——	——
Mean variation	0.82	3.56	10.48	2.7	——	——

### 2.2. Procedure

In this experiment, BigRun Teamx was used to monitor ECG signals, while the Kubios H.St system was employed to analyze heart rate variability (HRV). The Nordic Track T6.5S treadmill was used for gradient simulation, and the 6–20 Borg’s Rating of Perceived Exertion（RPE） subjective rating scale was utilized to measure participants’ perceived fatigue levels. Additionally, the H.Benchmark 2.0 reaction time training device was used to analyze participants’ attention levels.The experiment utilized individual resting-state data as a baseline to quantify relative changes in fatigue levels. However, the absence of an independent control group may limit the external validity of the results. Future studies will incorporate multi-group comparisons and long-term baseline tracking to further validate the model’s generalizability.

**Fig 1:**
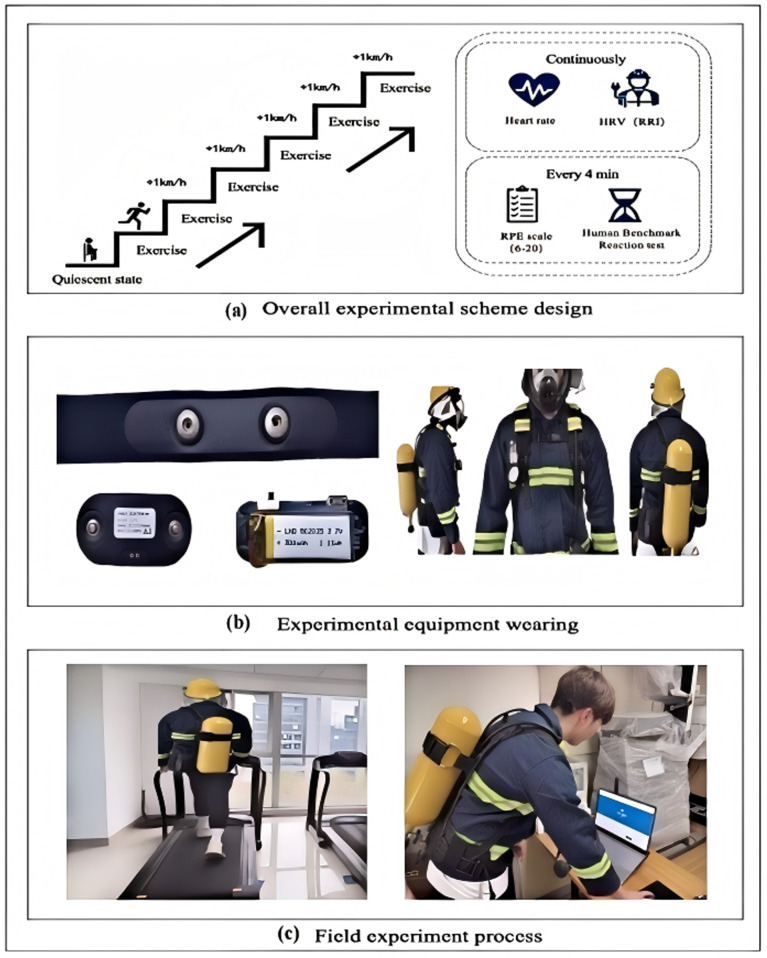
Experimental design and process.

During the field experiment, participants’ initial physiological data were measured before starting the tasks to establish individual baselines. This included recording ECG signals for 4 minutes while maintaining a resting state, conducting a brief fatigue self-assessment, and performing reaction time tests.The gradient exercise began with an initial speed of 4 km/h, with each stage lasting 4 minutes and the speed increasing by 1 km/h at each stage. At the end of each stage, participants immediately provided subjective fatigue ratings using Borg’s RPE scale and underwent simple reaction time tests (SRT). The detailed experimental process is shown in Fig 1.

### 2.3. Measures

#### 2.3.1. K-means clustering.

The Borg’s perceived fatigue values and reaction time data obtained from the experimental process were used as two feature values for K-means clustering analysis. The specific analysis process was performed using MATLAB 2016, and the detailed process was as follows:

(1) Creating a data set that includes 42 objects, each with two feature values X=[x_1,1_, x_1,2_, …x_1,42_, x_2,1_, x_2,2_, …x_2,42_], X=[6–20 Borg’s RPE, reaction time value].
(2) Randomly selecting K sample points as initial centroids, which are the center points (initial means) b_1_, b_2_…b_k_ of a cluster.
(3) Traversing each remaining sample point, calculating their Euclidean distance to K centroids, and put them into the cluster where the distance to the centroid is the smallest.

$$\begin{array}{c} d_{12}=\sqrt{{{(x}_{1}-x_{2})}^{2}+({y_{1}-y_{2})}^{2}} \end{array}$$ (1)

(4) Calculating the new centroids. In each iteration process, calculating the mean of all samples of each cluster, which is the new centroid of each cluster.

Repeat (3) and (4) until the centroids and the clusters do not change or reach the maximum number of iterations to obtain the classification results.

#### 2.3.2. Processing of operational fatigue evolution model’s output data based on entropy weight method.

The entropy weight method is used to solve the weight of each feature attribute in multiple attribute decision-making problems [8]. According to the size of feature information entropy to objectively assign weights to the feature, the greater the information entropy, the greater the degree of discreteness of that feature, and the more information it contains, the greater weight assigned to it [9]. The subjective data of 6–20 Borg’s rating of perceived exertion values and reaction time values were selected as the output feature data of the operational fatigue evolution model. The entropy weight method was applied to analyze the two features. By analyzing the diversity of perceived fatigue values and reaction time values, the higher the difference in feature value, the greater the degree of discreteness, indicating that the feature’s importance is higher, and it contains more information. The coefficients of the two features determined the weights through entropy weighting based on the degree of order of information contained. The specific process was completed through MATLAB programming.

(1) Data normalization processing. Each feature of the sample object is normalized one by one.

$$\begin{array}{c} x_{ij}^{\prime }=\frac{x_{ij}-x_{\min}}{x_{\max}-x_{\min}} \end{array}$$ (2)

$$\begin{array}{c} x_{ij}^{\prime \prime }=x_{ij}^{\prime }+0.001 \end{array}$$ (3)

(2) For one feature, calculate the proportion of all values taken by each sample under this feature.

$$\begin{array}{c} p_{ij}=\frac{x_{ij}^{\prime \prime }}{\sum_{i=1}^{n}x_{ij}^{\prime \prime }} \end{array}$$ (4)

(3) Calculate the entropy of each feature.

$$\begin{array}{c} e_{j}=-\frac{1}{\ln(n)}\sum_{i=1}^{n}p_{ij}\ln\left(p_{ij}\right) \end{array}$$ (5)

(4) Calculate the difference coefficient of each feature.

$$\begin{array}{c} g_{i}=1-e_{j} \end{array}$$ (6)

(5) Normalized the difference coefficient and calculated the weight of each feature.

$$\begin{array}{c} w_{j}=\frac{g_{j}}{\sum jg_{i}} \end{array}$$ (7)

#### 2.3.3. Construction of operational fatigue evolution model based on BP neural network.

BP neural network, also called backpropagation neural network, is a commonly used algorithm in artificial neural networks [10]. Its main framework consists of input layer, hidden layer, and output layer, with multiple neurons in each layer. A neuron is the basic unit of the BP neural network, which receives inputs from other neurons, weights them, and outputs them through the activation function. The training process of the BP neural network mainly relies on the backpropagation algorithm, which is implemented through gradient descent optimization algorithm. The basic idea is to update the model parameters along the negative gradient direction and minimize the loss function through iteration, ultimately achieving the goal of minimizing the loss function [11]. Because the values of various indicators of the collected personnel fatigue state are nonlinear and irregular data [12], while the BP neural network has excellent fault tolerance and generalization ability and can handle high-dimensional data and nonlinear problems, therefore, this machine learning algorithm is suitable for solving problems with complex internal mechanisms. The basic structure and working principle of the neural network.

Electrocardiogram is a reliable measurement method for evaluating fatigue status. The continuous depolarization and repolarization of myocardial cells in the heart produce electrocardiogram signals, which can reflect changes in heart rate and analyze heart rate variability by analyzing the interval between R and R points in the electrocardiogram signal wave. Studies have shown that electrocardiogram signals can well express the level of human fatigue and are one of the most important indicators commonly used to evaluate the level of central nervous system tension [13]. Heart rate (HR) is the number of heart beats per minute in the calm state of the human body, that is, the number of R-R intervals (or P-P intervals) in the electrocardiogram signal within one minute. Heinze et al. found that heart rate variability reflects the regulation of the tension and balance states between the sympathetic and parasympathetic nervous systems in the human body, which can reflect the psychological and physiological stress levels of the human body in different scenarios [14]. Heart rate and heart rate variability are two highly sensitive physiological indicators that can objectively reflect the level of fatigue and are easy to extract in electrocardiogram signal monitoring [15].

The 10 input features derived from heart rate (HR) and heart rate variability (HRV) metrics included:

- R-R interval (RRI),
- Heart rate (HR),
- Standard deviation of normal-to-normal intervals (SDNN),
- Root mean square of successive differences (RMSSD),
- Low-frequency power (LF),
- High-frequency power (HF),
- LF/HF ratio,
- Short-term variability (SD1),
- Long-term variability (SD2),
- SD2/SD1 ratio [16]

The 10 input features were collected and processed from the experiment are used as the input X [x_1_, x_2_, …x_10_] of the model, and the comprehensive evaluation index of operational fatigue (F) is used as the output Y1. The data is divided into a training set and a prediction set in a ratio of 9:1. A newff function is called to establish a trainable neural network object, and the transfer function of the hidden layer is set as tansig hyperbolic tangent function [17]. The transfer function of the output layer is set as purelin linear function, and the trainlm training function is called for prediction and training. The state of each layer node only affects the state of the next layer node. If the output value Y of the output layer does not match the expected value, the neural network threshold and weight are adjusted based on the prediction error, entering into the backpropagation, thus making the constructed BP neural network prediction output set approach the expected value [18]. The fatigue state prediction achieves the expected effect, and the error is within an acceptable range, the neural network prediction model building is completed, and finally, the prediction of firefighter fatigue index F is achieved.

## 3. Results and discussion

### 3.1. Fatigue level division based on K-means clustering analysis

**Fig 2:**
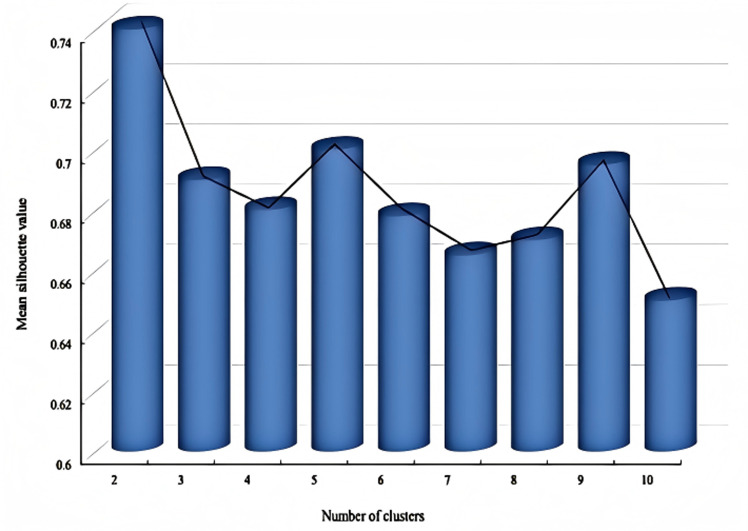
Silhouette value diagram.

The data is imported into the MATLAB platform for the K-means clustering process. According to the literature and experience, the clustering accuracy will be lower if the number of clusters is too small or too large. A code is written to give K from 2 to 10 clusters, Initialize the silhouette coefficient, traverse the distribution of different cluster coefficients under different K values, and repeat the clustering process ten times for each iteration. Calculate and output the silhouette coefficient graph of different cluster K values, as shown in Fig 2.

**Fig 3:**
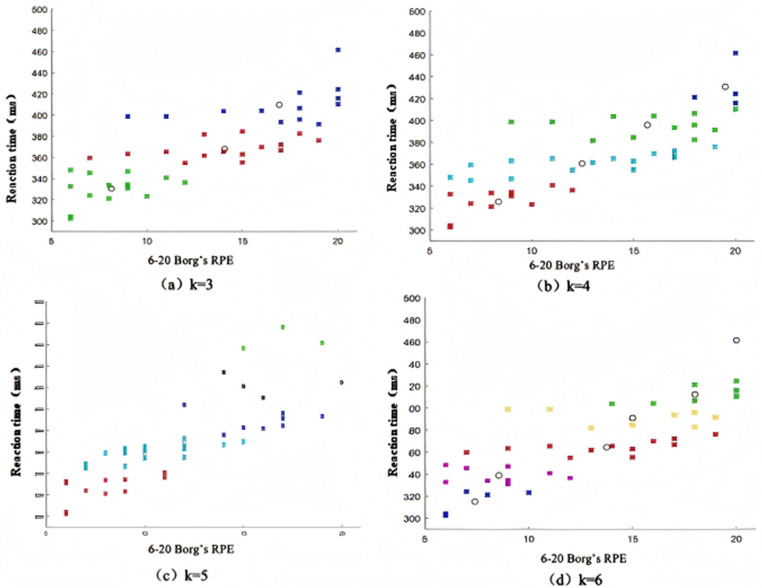
K-means cluster output.

The line graph shows that when the number of clusters K = 2, the silhouette coefficient is the highest and the clustering effect is the best. However, currently, dividing it into two categories does not meet the requirements of fatigue grading research. Therefore, further visual clustering analysis is carried out under the case of K = 3, K = 4, K = 5, and K = 6, and the clustering effect graphs under different K values are output, as shown in Fig 3. Considering the silhouette coefficient and clustering effect, the number of clusters is selected as 3, the silhouette coefficient of the cluster analysis is the largest, which represents the best classification effect. Therefore, K = 5 is selected as the number of fatigue state clusters. At the same time, based on the K-means clustering algorithm, the grading thresholds for y1 and y2 in the case of K = 5 are obtained, as shown in Table 2.

**Table 2:** Perceived Fatigue value and reaction time classification threshold.

classification	y1	y2
1	(0.00, 0.21)	(0.00, 0.18)
2	(0.07, 0.36)	(0.18, 0.33)
3	(0.21, 0.79)	(0.31, 0.58)
4	(0.57, 1.00)	(0.51, 0.88)
5	(0.79, 0.93)	(0.92, 1.00)

### 3.2. Calculation of comprehensive evaluation index F of occupational fatigue based on entropy weight method

According to the two characteristic data of reaction time and 6–20 Borg perceived fatigue values collected by experimental personnel in the experiment, the weight is scored for each characteristic through the entropy weight method, and the feature weight coefficient situation is finally obtained. Define a new variable F (Fatigue), establish an occupational fatigue comprehensive evaluation index as the fatigue grading indicator object, fatigue index F: F = 0.39R + 0.61B, where R is the reaction time, and B is the Borg.

The threshold values of each feature in Table 2 were adjusted with weighted coefficients to derive the classification thresholds for the comprehensive fatigue evaluation index FFF, as shown in Table 3. After comparison, it was observed that Level 1 and Level 2 significantly overlapped, and Level 4 and Level 5 were mutually inclusive. Therefore, the fatigue levels were ultimately consolidated into three categories: Level 1 (mild fatigue), Level 2 (moderate fatigue), and Level 3 (severe fatigue).

**Table 3:** Comprehensive evaluation index of occupational fatigue.

Classification	y1	y2	Fatigue
1	(0.00, 0.21)	(0.00, 0.18)	(0.00, 0.18)
2	(0.07, 0.36)	(0.18, 0.33)	(0.15, 0.30)
3	(0.21, 0.79)	(0.31, 0.58)	(0.27, 0.66)
4	(0.57, 1.00)	(0.51, 0.88)	(0.66, 0.89)
5	(0.79, 0.93)	(0.92, 1.00)	(0.87, 0.92)

**Level 1: Mild fatigue** (F∈[0,0.27): This phase indicates a state of relaxation to light fatigue, during which firefighters are required to quickly engage in firefighting and rescue operations.

**Level 2: Moderate fatigue** (F∈[0.27,0.66): This phase represents a rapid escalation in fatigue, considered a critical time for firefighting and rescue operations. During this stage, the autonomic nervous system (sympathetic/parasympathetic) and the vagus nerve are jointly regulating the body [19], keeping it in an active state with highly focused attention and agility.

**Level 3: Severe fatigue to exhaustion** (F∈[0.66,1.00): At this stage, the vagus nerve becomes dominant [20], and firefighters experience peak levels of accumulated fatigue as rescue operations continue. Attention and reaction speeds decline rapidly, requiring firefighters to promptly exit the fireground for rest and rotation.

**Fig4:**
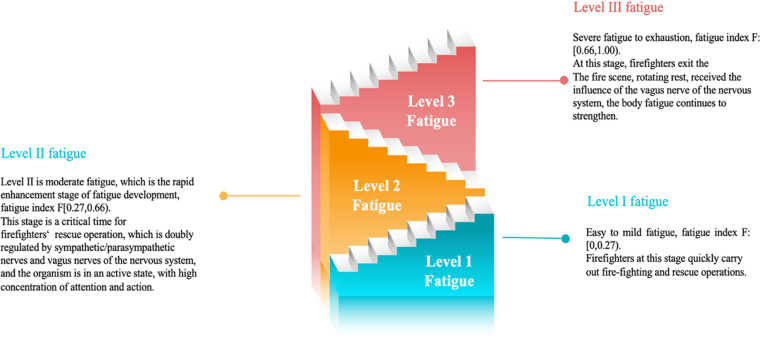
3-level classification model of firefighter fatigue.

The 3-level classification model for firefighter fatigue states is illustrated in Fig 4.

### 3.3. Construction of BP neural network model

**Fig 5:**
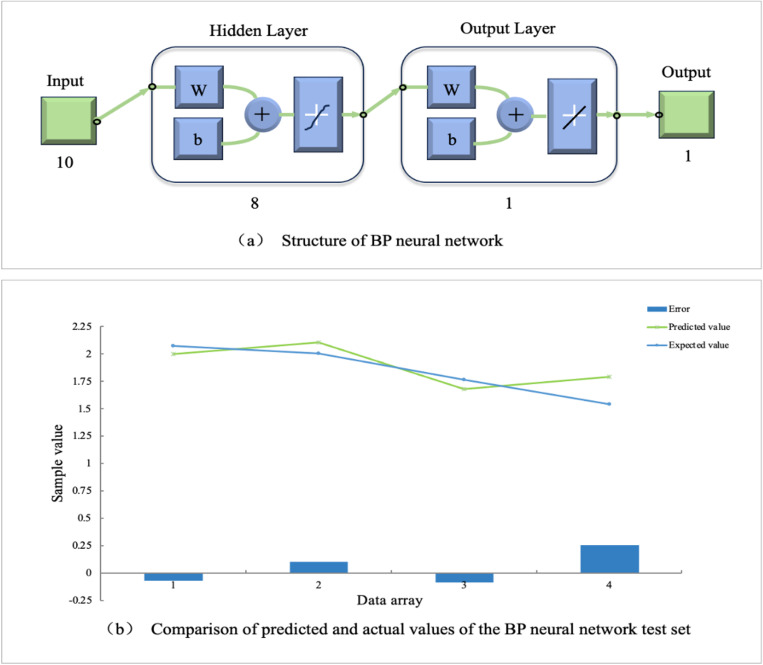
BP neural network structure and training interface.

Forty-two sample data were collected in the experiment, with each sample containing 10 input feature values and 1 output end. The training and prediction data were set at a ratio of 19:2. The number of input layer nodes was 10, the number of hidden layer nodes was 8, and the number of output layer nodes was 1. The training set was normalized to the range [-1,1] using the mapminmax function. The newff function was used to construct the neural network model, with the purelin activation function and gradient descent training. The maximum number of training times was set to 1000, the learning rate was 0.01, and the minimum error training goal was set to 0.00001. By running the MATLAB program with different numbers of hidden layer nodes, the node number was determined to be 8 and the neural network structure was 10-8-1, as shown in Fig 5.

**Fig 6:**
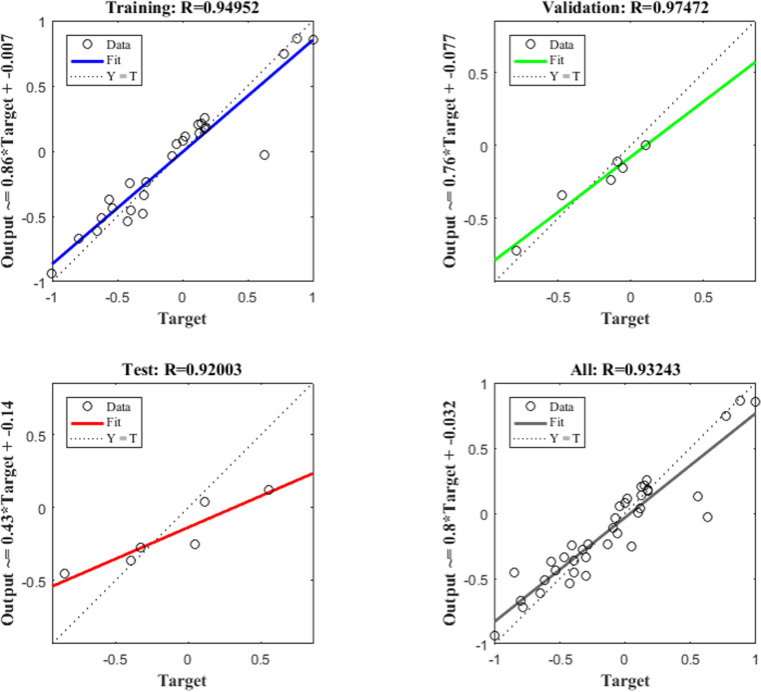
Fitting effect of the firefighter fatigue prediction model.

After training, the 10-8-1 model achieved the required prediction error. The hidden layer node number was 8, and the mean absolute error (MAE) was 0.12608, the mean squared error (MSE) was 0.021057, and the root mean squared error (RMSE) was 0.14511. The correct classification rates of the neural network model for the training set, validation set, and testing set sample data were 94.95%, 97.47%, and 92.00%, respectively. The overall prediction reliability was 93.24%, and the prediction effect of the model was good, as shown in Fig 6.

The fatigue prediction model developed in this study provides a technical foundation for real-time monitoring during fireground operations. For example, by using wearable devices (e.g., smart heart rate monitors, helmet sensors) to continuously collect firefighters’ ECG signals and reaction time data, combined with the model’s fatigue level output (F-value), dynamic alerts can be issued for severe fatigue states (F ≥ 0.66). This enables command systems to promptly rotate personnel. Additionally, the model’s predictions can be integrated with environmental parameters (e.g., temperature, smoke concentration) to optimize task allocation strategies and reduce the risk of operational errors caused by fatigue.

## 4. Conclusion

This study developed a dynamic fatigue classification and prediction model by integrating firefighters’ electrocardiogram (ECG) signals, Borg’s Rating of Perceived Exertion (RPE) scores, and reaction time data. The key conclusions are as follows:

(1) Scientific and Practical Fatigue Classification Model:Using K-means clustering and entropy weight method, firefighters’ fatigue states were classified into three levels. The classification thresholds align closely with autonomic nervous system dynamics (sympathetic/vagal modulation) and operational performance (e.g., slowed reaction speed), providing a quantitative basis for real-time monitoring.
(2) High Accuracy and Generalizability of the Prediction Model: The backpropagation (BP) neural network (10-8-1 architecture) demonstrated excellent performance on laboratory data (goodness-of-fit: 93.24%, MAE = 0.126). Its stability was confirmed via 5-fold cross-validation (mean MAE = 0.128), laying the groundwork for field applications in firefighting scenarios.
(3) Addressing Gaps in Dynamic Task Fatigue Research: Unlike existing models for static scenarios (e.g., drivers, dispatchers), this study pioneers the integration of multimodal data (physiological + subjective + cognitive) to address the dynamic demands of firefighting, offering a new paradigm for fatigue management in complex environments.

### Future directions

To advance the practical application and scientific rigor of this work, future research should prioritize the following objectives:

Field Deployment and Multidimensional Data Integration: Collaborate with firefighting agencies to deploy wearable monitoring devices (e.g., smart helmets, biometric sensors) in real-world rescue missions. This will enable the collection of multidimensional data, including environmental parameters (temperature, humidity, smoke density) and task-specific metrics (duration, load intensity), to calibrate and validate the model under dynamic operational conditions.

Real-Time Decision Support Systems: Develop an embedded warning system integrated into firefighters’ communication gear (e.g., headsets, visor displays) to provide real-time fatigue feedback. Such a system could alert command centers when critical fatigue thresholds (e.g., F≥0.66) are reached, facilitating timely personnel rotation and task reallocation.

Model Refinement via Historical Incident Analysis: Analyze historical fire incident reports and casualty statistics to establish empirical correlations between fatigue levels, mission success rates, and safety outcomes. These insights will refine model thresholds and enhance its predictive accuracy in high-risk scenarios.

### Limitations

Although this study has achieved certain advancements in fatigue classification and prediction methods for firefighters, the following limitations remain

#### Sample and experimental design limitations.

Small Sample Size: Only six healthy adult male participants were included, limiting the model’s generalizability.

Lack of Longitudinal Baseline Data: The experiment was conducted over a single time period, without collecting long-term physiological baseline data (e.g., resting heart rate, daily exercise load), which may affect the stability of fatigue classification.

Simplistic Experimental Conditions: The study was conducted in a controlled laboratory environment with constant temperature and humidity, failing to simulate real-world fireground conditions (e.g., high temperature, humidity, or smoke). This raises questions about the applicability of the results to actual operational scenarios.

#### Limitations in data collection and indicator selection.

Insufficient Physiological Metrics: Key indicators such as maximum oxygen uptake (VO₂max) and blood lactate levels were not included, potentially compromising the comprehensiveness of fatigue assessment.

Lack of Cognitive Function Evaluation: Only simple reaction time (SRT) was used to measure basic response speed, without assessing higher-order cognitive functions (e.g., inhibitory control via Stroop tests). This limits the ability to fully understand the impact of fatigue on decision-making.
